# Why Does Knocking Out NACHO, But Not RIC3, Completely Block Expression of α7 Nicotinic Receptors in Mouse Brain?

**DOI:** 10.3390/biom10030470

**Published:** 2020-03-19

**Authors:** Anish Deshpande, Remitha M. Vinayakamoorthy, Brijesh K. Garg, Jaya Prakash Thummapudi, Gauri Oza, Ketaki Adhikari, Aayush Agarwal, Parnika Dalvi, Swetha Iyer, Sarulatha Thulasi Raman, Vijay Ramesh, Akshitha Rameshbabu, Alexandra Rezvaya, Sneha Sukumaran, Sweta Swaminathan, Bhargav Tilak, Zhiyuan Wang, Phu V. Tran, Ralph H. Loring

**Affiliations:** 1 Department of Pharmaceutical Sciences, Northeastern University, Boston, MA 02115, USA; 2 Center for Neurobehavioral Development, Department of Pediatrics, University of Minnesota, Minneapolis, MN 55455, USA

**Keywords:** Protein folding, multi-subunit membrane protein assembly, receptor chaperone, alternate splice variants, antibody specificity, in vitro vs. in vivo effects

## Abstract

Alpha7 nicotinic acetylcholine receptors (α7nAChRs) are interesting not only because of their physiological effects, but because this receptor requires chaperones to traffic to cell surfaces (measured by alpha-bungarotoxin [αBGT] binding). While knockout (KO) animals and antibodies that react across species exist for *tmem35a* encoding the protein chaperone NACHO, commercially available antibodies against the chaperone RIC3 that allow Western blots across species have not been generally available. Further, no effects of deleting RIC3 function (*ric3* KO) on α7nAChR expression are reported. Finally, antibodies against α7nAChRs have shown various deficiencies. We find mouse macrophages bind αBGT but lack NACHO. We also report on a new α7nAChR antibody and testing commercially available anti-RIC3 antibodies that react across species allowing Western blot analysis of in vitro cultures. These antibodies also react to specific RIC3 splice variants and single-nucleotide polymorphisms. Preliminary autoradiographic analysis reveals that *ric3* KOs show subtle αBGT binding changes across different mouse brain regions, while *tmem35a* KOs show a complete loss of αBGT binding. These findings are inconsistent with effects observed in vitro, as RIC3 promotes αBGT binding to α7nAChRs expressed in HEK cells, even in the absence of NACHO. Collectively, additional regulatory factors are likely involved in the in vivo expression of α7nAChRs.

## 1. Introduction

Nicotinic acetylcholine receptors (nAChRs) play important physiological roles in the body, particularly in the brain, autonomic nervous system, and at neuromuscular junctions [1]. Seventeen known nAChR subunits co-assemble as pentamers in various combinations to form multiple receptor subtypes [2] and the process of how these receptors fold and assemble is not well understood. To date, several proteins have been identified as chaperones during nAChR assembly and are required for the surface expression of many nAChR receptor subtypes. At least two chaperones, Resistance to Inhibitors of Cholinesterase 3 (RIC3) and TMEM35A/Nicotinic Acetylcholine Regulator (NACHO) participate in folding, assembly and surface expression of the α7nAChR subtype as measured by the ability of cell surface receptors to bind alpha-bungarotoxin [3,4,5,6]. RIC3 was originally identified in a screen for mutations that allow the nematode C. elegans to survive after exposure to aldicarb, an acetylcholinesterase inhibitor [7]. RIC3 is highly conserved across animal species [8] and plays a critical role in regulating the assembly of the α7nAChR subtype as well as related serotonin 5HT3 receptor subtypes [2,6]. TMEM35A protein (Transmembrane protein 35A) was originally called TUF1 (for The Unknown Factor-1) [9]. Kennedy et al. prepared antibodies against TMEM35A (available as Sigma cat. # HPA048583) and generated a knockout (KO) animal [10]. These proved useful when David Bredt’s lab used an unbiased calcium influx screen, showing that TMEM35A is an important chaperone for α7nAChR functional expression [3,4]. Although TMEM35A is still the official gene name, Bredt’s group has renamed the protein NACHO. Hereafter, *tmem35a* and NACHO refers to the encoding gene and polypeptide, respectively. Similarly, RIC3 refers to the protein and *ric3* to the gene, while *chrna7* refers to the gene for the α7nAChR subunit. The *tmem35a* gene is located on the X chromosome of both mice and humans and is unrelated to *tmem35b*, a gene with little sequence homology found on other chromosomes (#1 in human, #4 in mice).

Unlike antibodies available for NACHO, we previously demonstrated that no suitable antibodies for Western blot (WB) analysis of mouse, rat and human RIC3 were available in 2013 [11]. Such a lack of working antibodies limits the investigation into the precise role of RIC3 in facilitating α7nAChR expression in mammalian species. More importantly, a lack of reliable antibodies can lead to inconsistent and non-reproducible results, which pose a serious problem in biological research. However, several new antibodies are now available on the market. We sought to determine their specificity across several species including human and mouse. We also investigated whether RIC3 splice variants and single nucleotide polymorphisms (SNPs) affect the ability of these antibodies to bind using in vitro systems. Additionally, a major hindrance to studying RIC3 effects on α7nAChR expression was the lack of a *ric3* KO animal model. We report here preliminary results using such a knockout.

Finally, many publications have shown that available antibodies for α7nAChRs are not acceptable for reliable Western blots [12,13,14,15]. Following our recent publication confirming these results [16], Synaptic Systems contacted us to evaluate a polyclonal rabbit-anti mouse α7nAChR antibody directed against amino acids 491-502, which are identical in human and rat α7nAChRs. This paper is a progress report on the usefulness of these molecular tools for determining the respective roles of NACHO and RIC3 in promoting receptor folding, assembly and cell surface expression of α7nAChRs. While addressing these specific objectives, our findings generate new questions about the interactions between NACHO and RIC3.

## 2. Materials and Methods

### 2.1. Plasmids

Mouse alpha7 in pCMV6 with a DDK (FLAG) tag was purchased from Origene (catalog MR224522). A stop codon between the mouse *chrna7* open reading frame and the DDK tag was introduced via PCR methods between the *Cla*I and *Mul*I sites. The open reading frame lacking a stop codon from human *hric3*(S-)-YN (Origene #SC112180, originally #TC112180 in reference [11]) with SNPs C130Y (rs55990541) and D352N (rs11826236) was subcloned into Origene pCMV6-myc-DDK vector using *Bgl*II and *Mlu*I sites in PCR primers (IDT, Coralville, IA, USA). Human *hric3*(S-)-HF (Origene #RC205179) in Origene pCMV6-myc-DDK vector with SNPs P57H and I165F was used as supplied and was the NCBI reference sequence for human RIC3 isoform 1 (NM_024557.2) until being replaced by NM_024557.3 in August 2011. Xenopus *xric3*(S+) in pcDNA3.1+/C-(K)DYK was synthesized by Genscript (#OAf03325) and used as supplied. Rat *ric3*(S-) in Invitrogen pRep4 plasmid (without a DDK tag) was used as described [11]. Human *hric3*(S-)-DDK in pcDNA3.1 was produced from human *ric3* transcript variant 1 (*hric3*(S+)-DDK in pcDNA3.1+ with a C-terminal DDK tagged vector; Genscript #OHu11533) by PCR methods, deleting the serine codon around the *Ppu*MI restriction site at position 503 in the open reading frame. Similarly, mouse *mric3*(S-)-DDK was produced from mouse *ric3* transcript variant 1 (*mric3*(S+)-DDK in pCMV6-entry vector; Origene #MR225825) by PCR inserting a silent *Bss*HII site at position 520 in the open reading frame and deleting the serine codon in the PCR primer. Human α7nAChR (also referred to in this paper by the gene name *hchrna7* when used in transfections) in pCI-Neo was a gift from Roger Papke and Clair Stokes. Human *tmem35a* was obtained encoding a chimeric protein with a myc-DDK tag from Origene (Cat. # RC209790 in pCMV6) and the open reading frame with a stop codon was subcloned into an episomal pRep9 plasmid (Invitrogen) modified to have blasticidin antibiotic resistance (P9KB, the final sequence is available upon request) between the *Kpn*I and *Xho*I restriction sites. Human *tmem35a* tagged with green fluorescent protein (GFP) in pCMV6-AC-GFP vector was also obtained from Origene (Cat. # RG209790). All DNA sequences (available on request) were confirmed by Sanger sequencing (Genewiz, Cambridge, MA, USA).

### 2.2. Reagents, Antibodies, and Cell Lines

α-Bungarotoxin (αBGT) was obtained from Biotoxins Inc., St Cloud, FL, and radioiodinated using iodogen (Pierce Chemical, Rockford, IL) as previously described [17]. RIC3 antibodies are listed in Table 1. Rabbit polyclonal anti-GAPDH (# PA1-988) was obtained from Thermo-Fisher Scientific, Waltham, MA. Secondary HRP-conjugated anti-rabbit IgG (# 7074), anti-mouse IgG (#7076) and anti-DDK (#2368) were purchased from Cell Signaling Technology and used at 1:1000 dilutions.

Rabbit antibody lot 8630 directed against amino acids 491–502 from mouse α7nAChRs was a gift from Carsten Schmidt (Synaptic Systems) and was given for evaluation. Rabbit antibody to NACHO was obtained from Sigma (Catalog # HPA048583).

Rat pituitary GH3 and GH4C1 cells were purchased from ATCC (catalog # CCL-82.1 & CCL-82.2, respectively) and grown in F12 medium (Fisher cat# SH30026FS) supplemented with 15% donor horse serum (DHS), 5% fetal bovine serum (FBS), and 0.5% penicillin/streptomycin (P/S, Fisher cat# ICN1670249) for GH3 or F10 (Fisher cat. # MT10-070-CV) medium supplemented with 10% FBS and 0.5% P/S (GH4C1 cells). N27 cells derived from rat midbrain neurons were purchased from the University of Colorado and grown in RPMI medium (Fisher cat# MT-10-040-CV) with 1 mM Glutamine supplemented with 10% FBS and 1% P/S. H9C2 cells derived from rat myocardium were generously provided by Dr. Ban-An Khaw (Northeastern University) and grown in DMEM medium (Fisher cat. # SH30243FS) supplemented with 10% FBS and 1% P/S. Human neuroblastoma cells SH-SY5Y were purchased from ATCC (Catalog # CRL-2266) and grown in 1:1 DMEM and F12 media supplemented with 10% FBS and 1% P/S. Human neuroblastoma line SH-EP1 cells were generously provided by Dr. Ron Lukas (Barrow Neurological Institute, Phoenix, AZ, USA) and grown in DMEM with 10% DHS, 5% FBS and 1% P/S. Human embryonic kidney cells, HEK293A, were purchased from QBiogene Inc. (Now a subsidiary of MP Biochemicals) and grown in DMEM supplemented with 10% FBS and 1% P/S. Mouse macrophage-derived cells (RAW 264.7 cells) were purchased from ATCC (Catalog # TIB-71) and grown in DMEM supplemented with 10% FBS and 1% FBS. All cells were maintained at 37 °C in humidified air supplemented with 5% CO_2_.

### 2.3. Cell Transfections and Western Blots (WBs)

To control for variations in expression while evaluating antibodies, *ric3* was cloned into pCMV6 or pcDNA3.1 vectors with Myc-DDK tags at the C-terminus. Human Embryonic Kidney cells (HEK293) were seeded in 6 well plates at 10^6^ cells/well. Cells grew overnight prior to transfection. Plasmid DNA samples were diluted using opti-MEM medium (Fisher cat. # 31-985-070) to a ratio of 2 µL X-tremeGene HP DNA transfection reagent (Sigma cat. # 6366236001) for every 1 µg of DNA and then incubated for three days following transfection. Separate transfections of red fluorescent protein (RFP) cloned into Invitrogen pREP4 plasmid served to evaluate transfection efficiency by fluorescence microscopy. Transfections in binding assays were done similarly in 24 well plates seeded at 2 × 10^5^ cells/well using transfection reagents in the same proportions/well but corrected for volume. When mixtures of DNA were used, half the DNA was *chrna7* and the other half was either mixtures of chaperone DNAs or RFP (used as a transfection control). WBs were performed as previously described by Garg and Loring [19] with minor modifications. In brief, RIPA buffer was used for to generate protein lysate (Cell Signaling Technologies Catalog # 9806) with the addition of 1 mM EDTA, 1 mM PMSF and 1x protease inhibitor cocktail (Halt protease inhibitor from Thermo-Fisher, Waltham, MA, USA). The Pierce BCA protein (Fisher # PI23235) assay kit allowed total protein quantification. Unless otherwise noted, 30 μg protein was used per well. As discussed in the results and supplemental information, other lysis buffers were used for WBs on brain samples.

### 2.4. [^125^I]-Labeled-αBGT Binding Assay

Radioactive binding assays were performed to detect surface α7nAChR expression as previously described [19]. HEK cells were plated at 2 × 10^5^ cells/well in a 24-well plate on day 1 and transfected on day 2. Then, ^125^I-αBGT binding assays were performed when cells were 80% confluent or after four days. Cells were incubated with 10 nM ^125^I-αBGT (unless stated otherwise) for 3 h in Hanks Buffered Saline (HBSS, Sigma cat# H6136) with 1% bovine serum albumin (BSA, Fisher cat#BP1600100) at 4 °C to measure total surface binding. Nonspecific binding was determined by the addition of 1 μM αBGT. After washing the cells three times in HBSS + BSA to remove unbound toxin, cells were lysed for 10–15 min on ice by the addition of 100 μL extraction buffer (0.5 M NaOH, 1% Triton X-100). Lysates were transferred into polypropylene tubes and counted for 1 min with a Packard Cobra gamma counter. Specific binding was determined as the mean of quadruplicate samples of total binding minus the mean of quadruplicate nonspecific binding. The associated errors represent the square root of the sum of the standard deviations for total and nonspecific binding squared.

### 2.5. Generation of ric3 KO and tmem35a KO Mice, Brain Dissections and Preparation of Mouse Macrophages

The *ric3* KO mouse was generated via cryogenic recovery from MMRRC strain B6; 129S5-Ric3tm1Lex/Mmucd (032542-UCD). This KO mouse was originally generated and subsequently donated to MMRRC by Genetech, Inc. [20]. Following cryogenic recovery, *ric3* KO mice were generated by heterozygous breeding into C57BL/6. Mouse genotypes were determined by PCR amplification of tail DNA using forward oligo (CTAAGAGGCAACAAGAGGCTG), reverse oligo (TGCTGCCCAAGGCCTTCTTGTC), and Neo-specific oligo (GCAGCGCATCGCCTTCTATC). This protocol produces two PCR products: 280 bp (WT) and 376 bp (KO).

The *tmem35a* KO mice were generated by breeding WT (X + Y) males to heterozygous (X+ X-) females. Male pups were either WT or KO (X-Y). Genotyping of *tmem35a* KO animals was performed using tail DNA and PCR amplification as previously described [10].

To isolate brain tissue, mice were killed with an intraperitoneal injection of Pentobarbital (10 mg/Kg) using an approved protocol. Following the removal of brain from cranium, hippocampus, thalamus, and cortex were dissected on an ice-cold metal block and flash frozen in liquid N_2_. Dissected tissues were stored at −80 °C until use. All protocols for generating or maintaining wild type, *ric3*, or *tmem35a* animals were approved by the University of Minnesota Institutional Animal Care and Use Committee (IACUC) protocol 1807-36127A. Similarly, all protocols for harvesting mouse peritoneal macrophages from C57BL/6 mice were performed as previously described [19] as approved by the Northeastern University IACUC using protocol 15-0522R. Both the University of Minnesota and Northeastern University’s animal care and use programs hold assurances with the Office of Laboratory Animal Welfare (OLAW) and are accredited by the Association for the Assessment and Accreditation for Laboratory Animal Care (AAALAC).

### 2.6. 125. I-α-Bungarotoxin (αBGT) Autoradiography

Mouse brains were cryoprotected in OCT compound (Fisher Scientific), frozen in −20C, and sectioned (20 µm) with a cryostat (Leica). Mouse brain sections mounted on glass slides (Superfrosted Plus, Fisher Scientific) were preincubated with or without nonradioactive blocking drugs (1 μM αBGT) at room temperature for 30 min in Tris-buffered saline (120 mM NaCl, 50 mM Tris, pH 7.4) with 2 mg/mL bovine serum albumin (TBS+BSA). The sections were then incubated for 3 h in 5 nM ^125^I-αBGT with or without the same blocking drug at room temperature in TBS+BSA, and then washed thrice for 30 min at 4 °C in TBS+BSA each. The slides were drained onto filter paper, air dried and then pressed against Perkin Elmer SR phosphor screens. After five days or up to three weeks exposure in a film cassette, the phosphor screens were processed on a Perkin Elmer Cyclone Phosphor Imager. Staining brain sections with hematoxylin after exposure allowed imaging using a Keyence microscope. Brain regions were identified in accordance with previous literature [21,22]. Pixel densities of autoradiograms were analyzed using NIH ImageJ.

## 3. Results

**Figure 1:**
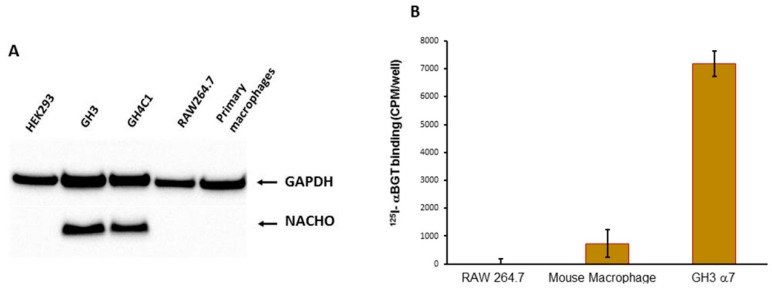
(**A**). Western Blot analysis of NACHO expression. GH3 and GH4C1 cells express endogenous NACHO, but mouse macrophage-like RAW264.7 cells, primary mouse macrophages, and HEK-293 do not. (**B**) In vitro ^125^I-αBGT binding assessments. Primary cultured macrophages and GH3 cells transfected with rat *chrna7* plasmid, but not RAW264.7 mouse macrophage-derived cells, showed ^125^I-αBGT binding.

We tested whether NACHO is required for surface expression of α7nAChR by correlating the presence of NACHO in various primary and transformed cell types with their ability to bind ^125^I-αBGT when *chrna7* is present. We previously reported both *chrna7* mRNA and αBGT binding in primary peritoneal macrophage cells from C57Bl/6 mice and this binding is absent in macrophages derived from *chrna7* KO mice [19]. We find that macrophages isolated from wild type animals do not express NACHO (Figure 1A), and yet express surface α7nAChR evident by ^125^I-αBGT binding (Figure 1B). We tested other cell lines (Figure 1A, Figure S1A) and found that endogenous NACHO is detectable only in human SH-SY5Y (Figure S1A), rat GH3, and rat GH4C1 cells (Figure 1A, Figure S1A). Together, these data suggest that NACHO is not a *sine qua non* for specific cell lines to fold, assemble, and traffic α7nAChRs to the cell surface.

**Figure 2:**
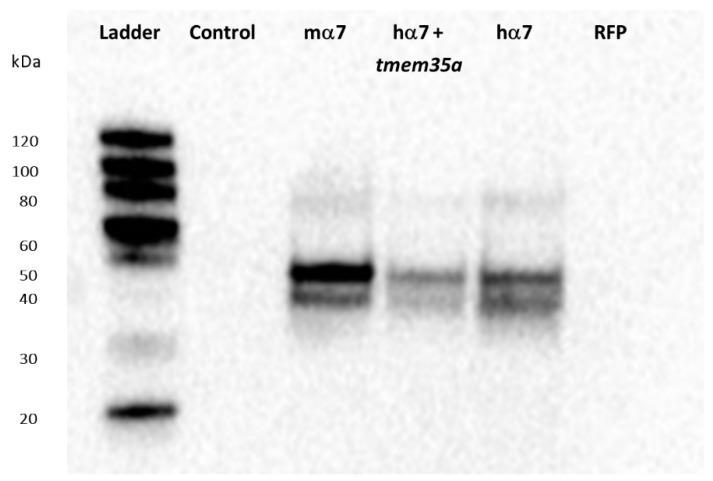
Western blot assessment of Synaptic Systems antibody against mouse α7nAChR. Antibody #8360 showed high specificity against human and mouse α7nAChRs and likely rat α7 given the identical C-terminal (antigen) sequence. Mouse α7 (100% *mchrna7*), human α7 (100% *hchrna7*), and human α7+NACHO (50% *hchrna7* +50% *htmem35a* DNA) transfected HEK cells showed bands corresponding approximately to the expected molecular weight (MW, ~55 KDa) and higher MW bands are also visible. Untransfected HEK-293 cells and cells transfected with Red Fluorescent Protein (RFP, a transfection control) showed no bands. Figure S2 shows that these constructs allowed surface ^125^I-αBGT binding when transfected into HEK cells, but not C-terminal FLAG tagged *mchrna7*.

We next tested the Synaptic Systems antibody against the mouse α7nAChR C-terminal using both human and mouse α7nAChRs expressed in HEK-293 cells (Figure 2). The antibody showed a smear at high molecular weights (~ 80 kD) and two bands between 50 and 40 kD. The two lower bands were not present in non-transfected cells (control) or RFP-transfected cells (RFP). Cells co-transfected with *tmem35a* and *hchrna7* at a 1:1 ratio showed a similar WB band pattern, but with decreased intensity, which was likely due to the decreased *chrna7* DNA concentration (Figure 2, *hα7+tmem35a*). The results showed that Synaptic Systems antibody is useful for WB analysis of human, mouse, and rat α7nAChRs in cultured cells.

**Figure 3:**
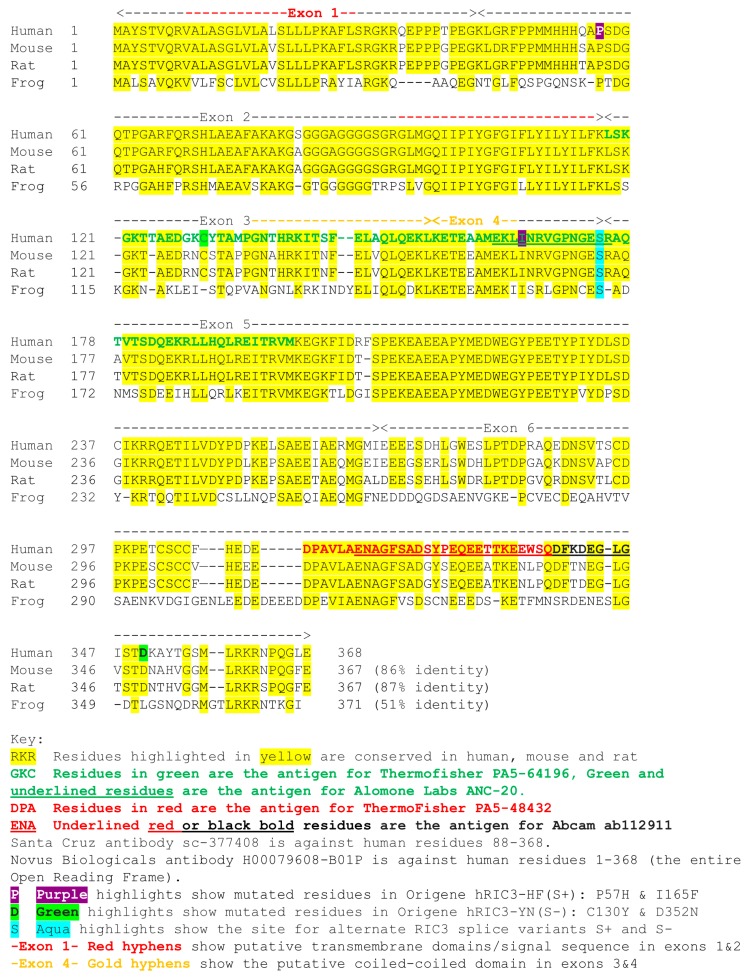
RIC3 sequences across species showing antibody antigens and mutations.

**Figure 4:**
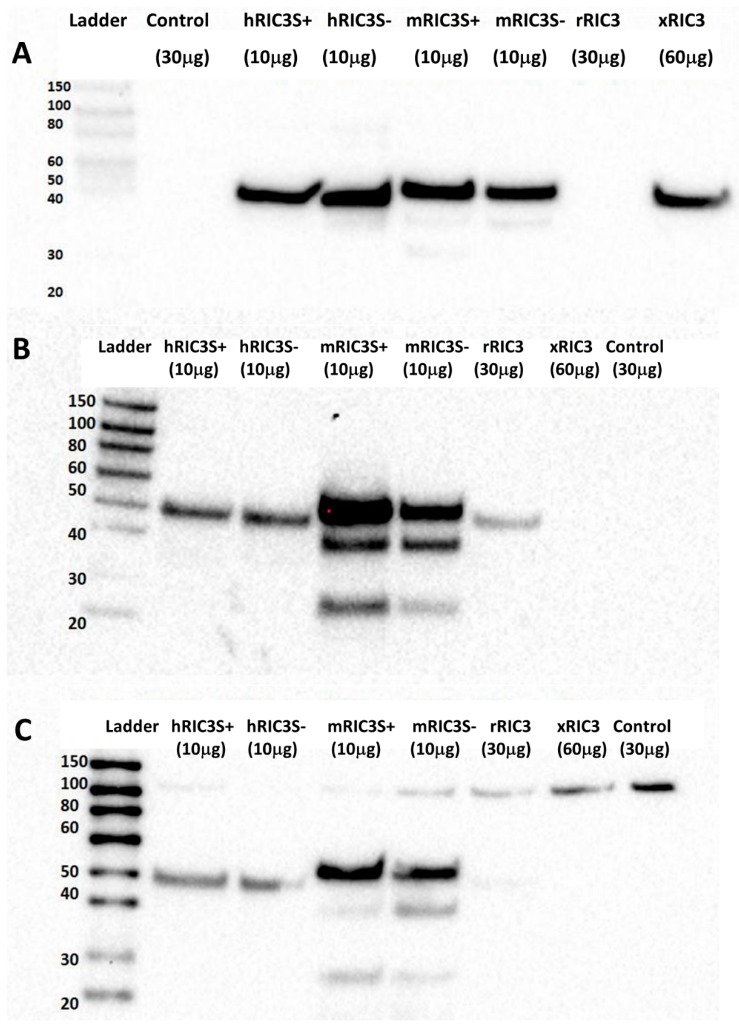
Assessment of RIC3 antibodies by Western blot. (**A**) Anti-DDK shows RIC3 expression efficiency following cell transfections. DDK–tagged RIC3s showed as single bands around 40 kD with considerable differences among different species. Rat RIC3 was not tagged with DDK. (**B**) Thermo-Fisher anti-hRIC3 PA5-64196 (1:1000) recognizes both splice variants of human and mouse RIC3 (with multiple bands) and weakly stains rat RIC3, but not Xenopus. (**C**) Alomone Laboratories anti-RIC3 antibody (ANC-020, 1:1000) showed a similar but weaker pattern and recognized both splice variants of human and mouse RIC3 (with additional bands) and weakly rat RIC3. A high MW (~100 kD) band is non-specific due to its presence in the control. Numbers in parentheses refer to the amount of protein added to each well to account for differences in protein expression between transfections.

A RIC3 sequence comparison among human, rat, mouse and xenopus showed the sequences that were used as antigens for six commercially available anti-RIC3 antibodies, as well as the location of the SNPs and splice variants present in our expression constructs (Figure 3). In addition, *ric3* gene exons are highly conserved across the same four species, with an ambiguous splice site between exons 4 and 5 that leads to the presence or absence of a single serine residue (denoted S+ or S-). Our DNA constructs (with the exception of rat *ric3*[S-]) were tagged with a DDK (FLAG) at the C-terminal, which was used to control for differential expression efficiency between different species (Figure 4A). Table 1 summarizes the properties of the six antibodies tested. All six antibodies recognized human RIC3, but only two showed staining for mouse and rat RIC3s. Thermo-Fisher anti-hRIC3 (PA5-64196) recognized both splice variants of human and mouse RIC3, and weakly rat RIC3, with a major band at approximately 40 kD (Figure 4B). This antibody did not recognize xenopus RIC3. Mouse RIC3 (both S+ and S-) showed additional smaller bands, suggesting proteolysis despite the presence of protease inhibitors. Alomone Laboratories ANC-020 anti-RIC3 antibody showed a similar band pattern and recognized both splice variants of human and mouse RIC3, and rat RIC3 (Figure 4C). Alomone Laboratories antibody also detected a high molecular weight non-specific band around 100 kD. Novus Biologicals H00079608-B01P anti-RIC3 weakly stained human, mouse and rat RIC3, and quickly lost its activity when stored with the other antibodies (Figure S3). Figure S4 shows the other three antibodies tested that recognized human RIC3, but not mouse or rat RIC3. Both Thermo-Fischer and Alomone antibodies that recognize mouse RIC3 showed reactivity to all SNPs in human *ric3* gene (Figure S5).

**Figure 5:**
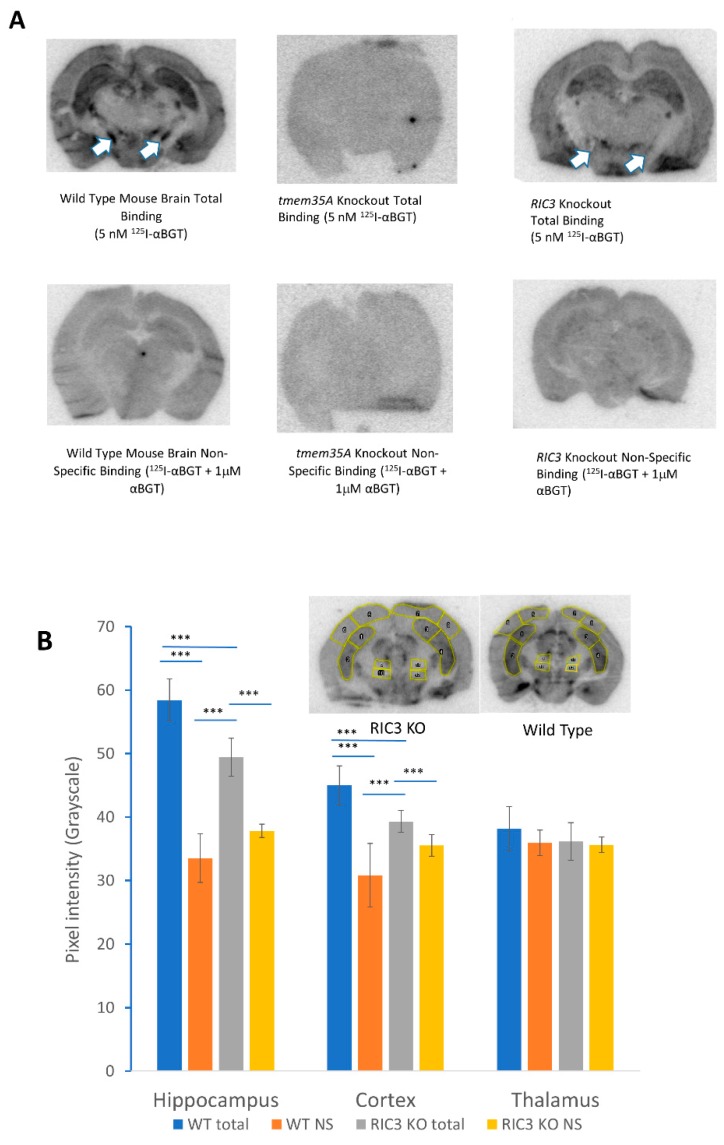
(**A**) Autoradiographic comparison of ^125^I-αBGT binding between wild type and KO animal brain slices. Top row shows total binding for wild type (left), *tmem35a* KO (middle) and *ric3* KO (right) brain sections. The bottom row shows corresponding non-specific binding. There was no specific binding in *tmem35a* KO, and significant loss of binding in specific brain structures in the *ric3* KO brains (arrows). (**B**) Autoradiographic analysis of ^125^I-αBGT binding using ImageJ. Significant loss of toxin binding was observed in the hippocampus and cortex of the *ric3* KO compared to the corresponding structures in wild type (WT) animals (Specific binding is the difference between total binding and non-specific [NS] binding). The insets show typical sections and the areas used for analysis over two sections per condition (N = 8 areas per brain region, with a medial and lateral area for each brain side times two sections). This analysis was done on one experiment comparing one animal per condition since the two experiments performed so far were done using different batches of ^125^I-αBGT with different specific activities and slightly different exposure times and are not easily comparable. Error bars represent standard deviations. *** *p* > 0.001, (**p < 0.01, * *p* < 0.05) by single factor ANOVA.

*ric3* KO mice showed no overt phenotype. Figure 5A shows the results of a preliminary autoradiographic analysis of ^125^I-αBGT binding to brain slices from wild type, *tmem35a* KO and *ric3* KO (N = 2/genotype) animals. In one experiment, hippocampal and cortical (coronal) sections of *ric3* KO mice showed a significant decrease of specific αBGT binding compared to wild type mice. Other smaller brain structures might have reduced toxin binding in *ric3* KO mice (Figure 5A arrows). Analysis of these structures will be pursued in future studies using high-resolution images. In this experiment, the examination of pixel intensity using ImageJ indicated that *ric3* KO brain slices have approximately 50% less toxin binding in hippocampus and a significant loss of binding in the cortex compared to wild type animals (Figure 5B). However, these results were not replicated in a second experiment (Figure S6), suggesting that the effects of RIC3 on α7nAChR expression in brain may be subtle. In contrast, *tmem35a* KOs (Figure 5 and Figure S6) showed a complete loss of ^125^I-αBGT binding in any brain regions, confirming previous findings by Gu et al. [3,4]. Collectively, *ric3* KO showed little effect compared to *tmem35a* KO on α7nAChR expression in mouse brain.

**Figure 6:**
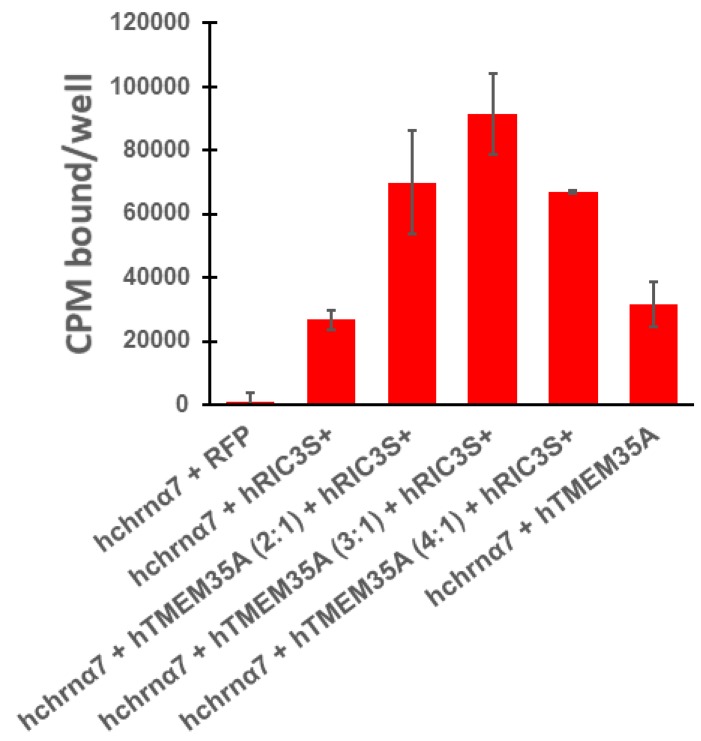
The absence of NACHO in HEK cells has no effect on the ability of RIC3 to promote surface human α7nAChR expression, and the effects of the two chaperones are synergistic when expressed together. Binding assays in 24-well plates were performed as indicated in methods. Total cDNA in transfections was constant, with *hchrna7* DNA (0.15 μg/well) that was equaled the sum of *htmem35a* and *hric3* cDNA or RFP DNA (0.15 μg/well RFP DNA in transfection controls). The ratio of 3 parts *htmem35a* cDNA to 1 part *hric3* cDNA (e.g., 0.11 µg *htmem35a* and 0.04 µg *hric3*/well) produced the highest surface α7nAChR expression in HEK cells. In all 4 experiments, the combined effects were more than additive. In experiments where RIC3 or NACHO was the only chaperone, surface α7nAChR expression was comparable between these two chaperones as shown.

These in vivo results contrast sharply with previous in vitro results. Gu et al. [3,4] report that RIC3 and NACHO act synergistically in HEK cells to promote surface α7nAChR expression as measured by electrophysiological recording and fluorescent αBGT binding. However, they did not demonstrate the effect of varying *ric3* and *tmem35a* cDNA ratios on surface α7nAChR expression. Alexander et al. [23] demonstrated that different ratios between *ric3* alone and *chrna7* sufficiently induce differential surface α7nAChR expression, with high *ric3* to *chrna7* ratios causing internal receptor aggregation and retention, resulting in an inverted U-shaped expression curve. Similarly, Ben-David et al. [24] noted an inverted U-shaped expression curve with varying amounts of mouse *ric3* cRNA injected with a fixed amount of *chrna7* cRNA into oocytes. These data suggest that the ratios between the three genes (*ric3*, *tmem35a*, and *chrna7*) will be important factors determining total surface receptor expression. We investigated the effects of maintaining *chrna7* level equal to the sum of the two chaperones in HEK cells (Figure 6). RIC3 promoted surface α7nAChR expression in HEK cells as evident by αBGT binding even with the absence of NACHO expression (Figure 6). The effects were synergistic as a 3:1 *tmem35a* to *ric3* ratio produced a threefold greater response than either gene alone.

We next tried to show the presence of NACHO, RIC3 and α7nAChR in hippocampal lysates of wild type, *tmem35a* KO and *ric3* KO animals. In wild type brain lysates, Sigma anti-NACHO shows appropriate bands as described [10], but not RIC3 (Thermofisher PA5-64196) or α7nAChR antibodies. Despite attempts using different lysis buffers, including RIPA, RIPA enhanced with 1% triton X100 and the buffer used by Kennedy et al. [10] for NACHO (10 mM Tris, pH 7.4, 100 mM NaCl, 1 mM EDTA, 1 mM EGTA, 1 mM NaF, 20 mM Na_4_P_2_O_7_, 2 mM Na_3_VO_4_, 1% Triton X-100, 10% glycerol, 0.1% SDS, 0.5% deoxycholate), none has worked so far for RIC3 or α7nAChR. These results indicate technical or biological challenges (e.g., post-translational modifications, too low expression levels). Based on the detection of the endoplasmic reticulum marker calnexin and NACHO (Figure S7) using an extraction protocol from Gu et al. [3], it is unlikely that technical difficulty was the source of the negative results. The available in situ hybridization data in the Allen Mouse Brain Atlas [21] suggests the rank order for mRNA concentrations in mouse cortex is calnexin >> *tmem35a* > *chrna7* > *ric3*. These data suggest that low levels of α7nAChR and RIC3 in mouse brain are likely limiting factors for antibody detection.

## 4. Discussion

Gu et al. and Matta et al. [3,4] proposed that NACHO is a master regulator required for α7nAChR folding, assembly and expression on cell surfaces. However, we find the presence or absence of NACHO in various cell lines does not necessarily correlate with the ability of the cells to support surface α7nAChR expression (Figure 1, Figure S1). In particular, primary mouse peritoneal macrophages do not require NACHO to express surface α7nAChR evident by αBGT binding (Figure 1), which is not detected in macrophages from *chrna7* KO animals [19]. Previous publications report difficulties showing functional α7nAChR ion channels in macrophages or similar cell types (reviewed in [19] but see [25]). As such, it has been proposed that these cells express metabotropic rather than ionotropic α7nAChRs [26]. We also tested other cell lines (Figure S1A) and found that SH-SY5Y, not SH-EP1, cells express NACHO. While some studies showed that SH-SY5Y cells endogenously express surface α7nAChR, we do not find this (see Figure S1A for further discussion). Mulcahy et al. [27] report the SH-EP1 cell line lacks RIC3, but is capable of αBGT binding when transfected with *chrna7*, and this binding does not change much when transfected with both *chrna7* and *ric3*. Here, we show that this cell line also lacks NACHO (Figure S1A). Taken together, these findings suggest additional factors regulating surface α7nAChR expression in these cells.

Others (and we) have previously demonstrated the problematic nature of available antibodies against α7nAChR [12,13,14,15,16]. These antibodies failed to generate reproducible results, which is a serious problem in biological research [28]. An ideal antibody raised against a protein will specifically recognizes its epitope on the target protein and nothing else. On this basis, the results with the Synaptic Systems antibody are encouraging. This antibody showed little non-specific binding in Western blots and the antibody recognizes receptors across multiple species using cell culture models. However, to date, we have not been able to establish its utility for α7nAChR immunohistochemistry in mouse brain.

In terms of RIC3 antibodies, antibodies that recognize homologous proteins between species are useful to detect differential protein expression across anatomical locations and cell types, while those that fail to recognize protein splice variants or SNPs may give an incomplete expression profile of a target protein. The production of antibodies often involves the selection of specific peptides as antigens. Linear protein epitopes normally consist of sequences of six to nine amino acids and conformational epitopes are often considered as linear epitopes brought together by the three-dimensional structure of proteins [29]. However, the actual binding contacts between an antibody and its protein epitope may involve only a small subset of amino acids within linear epitope sequences. Most of the RIC3 antibodies used in this study were raised against peptides, which suggests the possibility of multiple linear epitopes distributed over the length of the peptide (e.g., all polyclonal antibodies, except the Santa Cruz monoclonal antibody). In addition, besides the alpha helices in the transmembrane domain(s) and the coiled-coiled domain, RIC3 is a largely disordered protein [30], suggesting that antibody recognition should be based primarily on the linear amino acid sequences. However, Koperniak et al. [11] found that antibodies against human and rat RIC3 available in 2013 were highly dependent on protein conformation and that heat denaturation destroyed all binding. Therefore, we cannot rule out a contribution of protein conformation to recognition of RIC3 epitopes by antibodies.

In order to normalize for differences in transfection efficiencies or codon usage artifacts for RIC3 expression, we primarily used C-terminal Myc-FLAG tagged versions of human, mouse and xenopus. Presumably, the tag would not interfere with antibody binding. Wang et al. [31] demonstrated that C-terminal Myc or GFP tagging does not interfere with full-length mouse RIC3 activity. RIC3 exists in various isoforms due to alternate splicing. Alternate splicing is commonly believed to expand the number of functional proteins from a fixed genome [32], but considerable controversy exists whether many splice variants are truly functional or instead represent splicing errors (e.g., [33]). Antibodies that discriminate between splice variants would be highly useful to help settle this question. NCBI reference sequences (RefSeq) lists nine isoforms (*a, c, e, f, g, h, i, j, k*) of human *ric3* (Gene ID: 79608) based on the lettering scheme of Seredenina et al. [34]. Isoform *a* corresponds to full-length *hric3S+* and isoform *c* corresponds to *hricS-*. All the other isoforms are missing various exons, parts of exons, or contained insertions. These differences make it difficult to detect all splice variants with a single antibody. For instance, the first twelve amino acids (EKLINRVGPNGE) of the fourteen amino acid peptide used to produce the Alomone Laboratories antibody are present only in isoforms *a, c*, and *i*, even though the coding sequence is exclusively in exon 4. In contrast, the sequence of the 29 amino acid antigen from exon 6 used to produce ThermoFisher PA5-48432 is represented in seven of the nine human isoforms. However, the first twelve amino acids in the Alomone immunogen are represented in all three isoforms listed in RefSeq in mouse RIC3 (Gene ID: 320360). Ben-David et al. report a fourth mouse isoform (RIC3-TM, [35]) consisting of only exons 1 and 2 with an alternate C terminus and this isoform has no overlap with the shorter peptides used to produce the antibodies studied here. Based on sequences, the only antibodies we tested that could recognize RIC3-TM were the Santa Cruz monoclonal and the Novus Biologicals polyclonal antibodies. We found that the former did not recognize full-length mouse RIC3 that includes exons 1 and 2. Further work will be required to determine if the Novus antibody recognizes the mouse RIC3-TM isoform. Ben-David et al. [35] found mRNA for RIC3-TM in mouse brain and suggest it may have different functional effects from the full-length RIC3. These findings underscore the importance finding an antibody that recognizes mouse RIC3-TM. Nevertheless, Thermofisher/Invitrogen PA5-64196 and Alomone Labs ANC-020 should be useful for detecting the major mouse, rat and human RIC3 isoforms.

Gu et al. [3] and Matta et al. [4] propose that NACHO acts as a master regulator for folding and assembly of many types of nicotinic receptors including α7nAChRs. Several observations form the basis of this hypothesis for α7nAChRs: (1) *tmem35a* KO completely loses αBGT binding in mouse brain (which we confirmed in Figure 5 and Figure S6) and the results were similar to those seen for *chrna7* knockouts [36]; (2) RIC3 works synergistically with NACHO in heterologous expression systems such as HEK cells (which we confirmed in Figure 6); (3) *tmem35a* KO does not change *ric3* mRNA expression (Matta et al. Figure S1B [4]). Similarly, we found that hippocampal transcriptome of *tmem35a* KO mice showed no difference in *ric3* mRNA expression (data not shown). However, these results leave unanswered whether NACHO loss of function affects the ability of brain cells to express α7nAChR subunits or RIC3 proteins. Also, if RIC3 works synergistically with NACHO in vitro, why does knocking out *tmem35a* prevent RIC3 from being a functional chaperone in vivo? An alternative hypothesis to explain the data is that NACHO inhibits the action of an unidentified factor or that the lack of NACHO blocks the action of RIC3 through a mechanism, which is not found in heterologous in vitro expression systems. In this regard, as illustrated in the graphical abstract, NACHO is needed for functional RIC3 in brain cells. Using the new tools we are developing, we can start to address these issues. However, it is critically important to show that RIC3 protein is present in the brains of *tmem35a* KO and wild type animals.

Preliminary autoradiographic analysis of ^125^I-αBGT binding to mouse brain sections confirms that knocking out *tmem35a* lost virtually all αBGT binding as previously shown by Gu and Matta et al. [3,4]. In contrast, knocking out *ric3* causes variable effects depending on brain regions, but exact analysis of this phenomenon requires overcoming several technical hurdles. For example, the phosphor imaging sheets saturate and show a non-linear response to radioactivity. This tends to emphasize non-specific binding relative to specific binding, leading to high backgrounds. A standard curve of varying amounts of known radioactivity is necessary to convert differences in average pixel intensity to fmoles of bound αBGT per mm^3^ tissue. Because of this and other factors, such as differences in ^125^I-αBGT specific activity and age at the time of exposure, it is not possible to compare results exactly between different autoradiographic experiments at present. Also, we need better techniques to study small brain structures, to register brain regions between different animals, and to align the histological images of the brain sections with autoradiograms. Whiteaker et al. found many small brain structures in their autoradiographic analysis of ^125^I-αBGT and tritiated methyllycaconitine binding in mouse brain [37]. It may be necessary to use serial cryosectioning to make sure that small brain structures are present across several sections in comparisons between wild type and KO animals. None of these problems are insurmountable. In this study, we concentrated on large brain structures such as hippocampus and cortex due to their accessibility. However, even with this small sample size it is clear that knocking out *ric3* has less effects on α7nAChR expression than knocking out *tmem35a.* These data raise new questions about the interaction of these two chaperones. Measuring the relative amounts of mRNA and respective polypeptides for the two chaperones will be essential for determining why specific chaperone loss-of-function results in differential effects in brain.

Overall, the present data supports the conclusions of Gu et al. [3] and Matta et al. [4] that NACHO is a master regulator of α7nAChR folding, assembly and expression in mouse brain. What remains perplexing is the striking difference between the actions of NACHO and RIC3 in vivo vs. in vitro. Is the in vitro data an artifact of ectopic chaperone overexpression in HEK cells? Or, does NACHO in vivo regulate other unknown proteins or intracellular processes that impact receptor expression in ways not yet appreciated?

## 5. Conclusions

We found antibodies for RIC3 and α7nAChR that work well across mouse and human receptors for cells in culture, and may be adaptable for use in vivo. Knocking out RIC3 has subtle effects on αBGT binding to mouse cortex and hippocampus. In contrast, knocking out NACHO causes loss of virtually all αBGT binding. This latter observation led to the hypothesis that NACHO is required for all α7nAChR-dependent αBGT binding. As such, we find a contrary example that primary mouse macrophages express functional α7nAChR in the absence of NACHO, suggesting a NACHO-independent mechanism in specific cell (immune) types. In addition, the dramatic effects of knocking out NACHO in vivo are not comparable to experiments in vitro, as RIC3 works as a α7nAChR chaperone in HEK cells in the absence of NACHO. These results suggest that other regulatory factors may also be involved.

## Figures and Tables

**Table 1:** RIC3 antibodies used in this paper.

Primary Antibody *	Company	Catalog Number	Lot Number(s)
Anti-Human RIC3	Abcam	ab112911	GR99507-5
Anti-Human RIC3 **	Santa Cruz Biotechnology	sc-377408	H3117
Anti-Human RIC3	Thermofisher	PA5-48432	SF2408204A
Anti-Human RIC3	Thermofisher/Invitrogen	PA5-64196	SL2490062C, TE2576142A
Anti-Mouse RIC3 ***	Alomone Labs	ANC-020	ANC020AN0125
Anti-Human RIC3 ****	Novus Biologicals	H00079608-B01P	H6291

* Immunogen sequences are shown in Figure 3; ** A mouse monoclonal antibody, all others are rabbit polyclonal antibodies; *** Antibody designed against mouse RIC3 using the immunogen sequence that is identical in rat and human; **** Antibody against full-length human RIC3 and is reported to cross react with Chinese hamster and xenopus RIC3 [18].

## Supplementary Materials

The following are available online at https://www.mdpi.com/2218-273X/10/3/470/s1, Supplementary information for Deshpande et al. including Figures S1–S7.
