# Supplementary material for: Why Does Knocking Out NACHO, But Not RIC3, Completely Block Expression of α7 Nicotinic Receptors in Mouse Brain?
Source: Biomolecules. 2020 Mar 19;10(3):470. doi: 10.3390/biom10030470 (PMC7175337; doi:10.3390/biom10030470)
Supplement: Supplementary file 1 [file biomolecules-10-00470-s001.pdf]

Supplementary information for Deshpande et al., **Why does knocking out NACHO but not RIC3, completely block expression of α7 nicotinic receptors in mouse brain?**

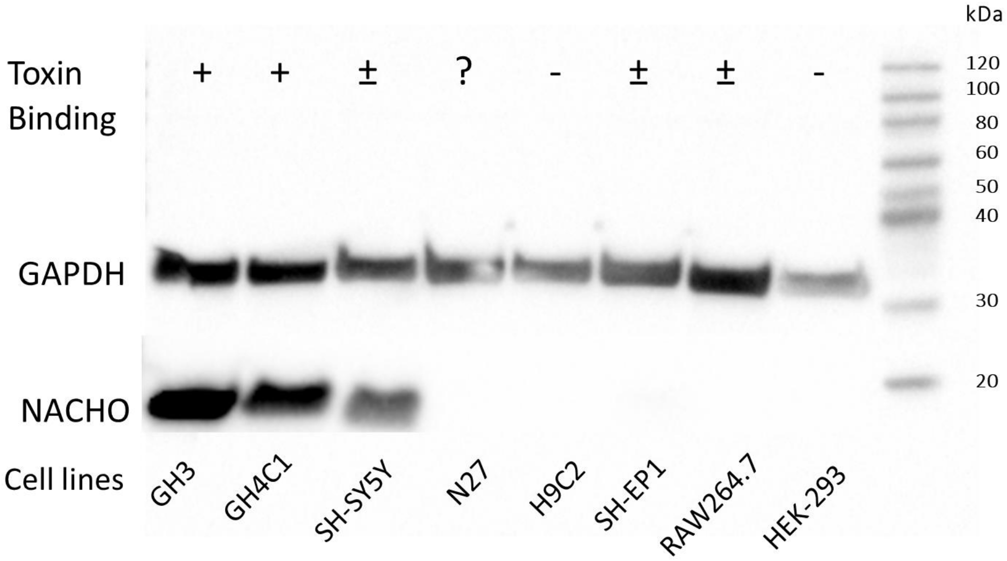

Figure S1A: Toxin binding in cell lines does not necessarily correlate with the presence of NACHO detected by Western blots. GH3 cells showed the highest NACHO expression, followed by GH4C1 (in some experiments similar amounts to GH3), with less NACHO in SH-SY5Y cells. No detectible NACHO were found in the other cell types tested. This figure is a composite of two blots. Key to symbols for reported toxin binding: (+) Surface toxin binding if endogenously expressing α7nAChRs or transfected with *chrna7* in the absence of added chaperones, (-)no surface toxin binding after transfection with *chrna7* without adding chaperones, (±) conflicting reports about surface binding, (?) no report in the literature and not tested. Sample citations for assigning the symbols (See commentary below for details):

1. GH3 + Fuerbach et al. [37]
2. GH4C1 + Koperniak et al. [11]
3. SH-SY5Y + Lukas et al. [38], - Hangqing Lin, unpublished data
4. N27 ? No literature found, not tested
5. H9C2 –Figure S1B
6. SH-EP1 + Peng et al. [39], - Koperniak et al. [11]
7. RAW264.7 + Liu et al. [40], - Figure 1A
8. HEK293 -Williams et al.[41]

Commentary: GH3 ([37] and Figure 1) and GH4C1 cells (e.g. [11]) are well established to express surface αBGT binding when transfected with *chrna7*. On the other hand, SH-SY5Y cells were the source for cloning human *chrna7* [42] and based on the presence NACHO in these cells (albeit lower than observed in GH3 or GH4AC1 cells), one might expect significant levels of endogenous surface αBGT binding. Some investigators reported significant levels of αBGT binding to SH-SY5Y cells [39] including forced expression of exogenous *chrna7* [43,44]. In our hands, SH-SY5Y cells do not show significant surface toxin binding (Hangqin Lin, unpublished; similar to untreated controls in figure 6A of reference [45]) and transfection with *chrna7* had only a modest effect compared with other cell lines. Therefore, we assigned a (±) designation to SH-SY5Y cells. We tested N27 cells derived from rat midbrain neurons, but there are no studies showing these cells with surface toxin binding even with forced expression of exogenous *chrna7*. H9C2 cells derived from rat myocardium did not express surface α7nAChRs when transfected by *chrna7* alone, but earned a (–) designation by readily expressed surface toxin binding when co-transfected with *chrna7* and human *tmem35a-eGFP* (Figure S1B). Similarly, HEK 293 cells lack NACHO (in agreement with [3]) and cannot express surface α7nAChRs when transfected by *chrna7* alone but readily express surface receptors when co-transfected with *chrna7* and *ric3* [41] or *tmem35a* (Figure 6 or [3,4]). Peng et al. [39] reported the SH-EP1 cells support surface toxin binding upon inducible *chrna7* transfection. Mulcahey et al. [26] also report significant toxin binding to *chrna7* transfected SH-EP1 cells and that adding RIC3 did not enhance binding significantly. We reported similar but low inducible surface expression when infected with adenovirus bearing *chrna7* [46]. However, later our SH-EP1 cells required co-transfection with *ric3* to support significant toxin binding [11]. It is possible that the original cell line did express NACHO, which was progressively lost following repeated passages. Based on the data we have available, we awarded a (±) designation to SH-EP1 cells and the role of NACHO in surface expression is unknown. RAW264.7 cells derived from mouse macrophages do not express NACHO in Figure 1 but are reported to bind αBGT [40, 47], although we failed to find significant surface αBGT binding (Figure 1B) leading to a (±) designation. Further, mouse macrophages express surface αBGT binding (Figure 1B) that is not present in macrophages from α7 KO animals [19] but like RAW264.7 cells, lack NACHO expression (Figure 1A). Therefore, although the data is somewhat ambiguous, NACHO expression might not be required for surface α7nAChRs in various cell lines or primary cultures, suggesting a yet unidentified chaperone.

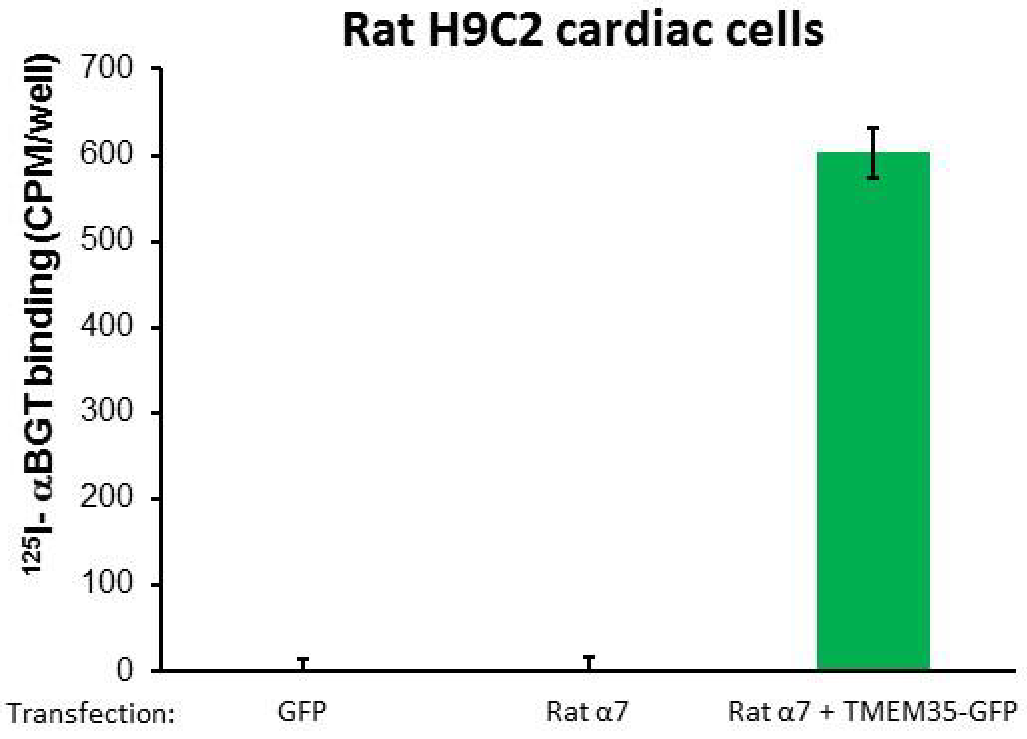

Figure S1B. Sample Binding Data for rat H9C2 cells: Human *tmem35A*-GFP was transfected into rat H9C2 cells together with rat $\alpha 7$ nAChR plasmid. This combination causes significant surface $\alpha 7$ expression measured by 125I-αBGT binding. Transfection with rat $\alpha 7$ or GFP alone causes no surface expression. Plasmids for GFP and rat $\alpha 7$ nAChR are as previously described [19], and TMEM35-GFP is human *tmem35a* in pCMV6-AC-GFP vector obtained from Origene (Cat. # RG209790).

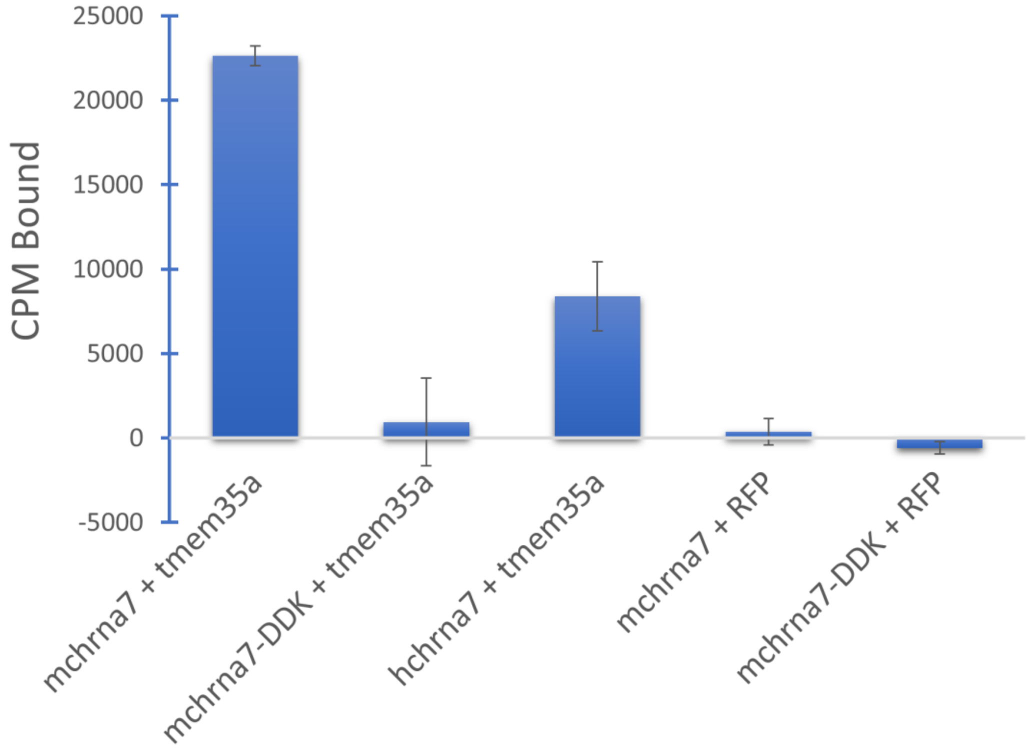

Figure S2. HEK293 cells transfected with mouse (*mchrna7*) or human (*hchrna7*) α7nAChR together with mouse *tmem35a* (1:1 ratio) showed significant surface 125I-αBGT binding, whereas cells transfected with DDK-tagged mouse α7nAChR plus mouse *tmem35a* failed to show significant binding. RFP was included as a transfection control in two other conditions. Besides confirming that both *mchrna7* and m*tmem35a* constructs are functional, these data suggest that the DDK sequence interferes with surface expression and thus a C-terminal FLAG tag for α7nAChR would not be useful.

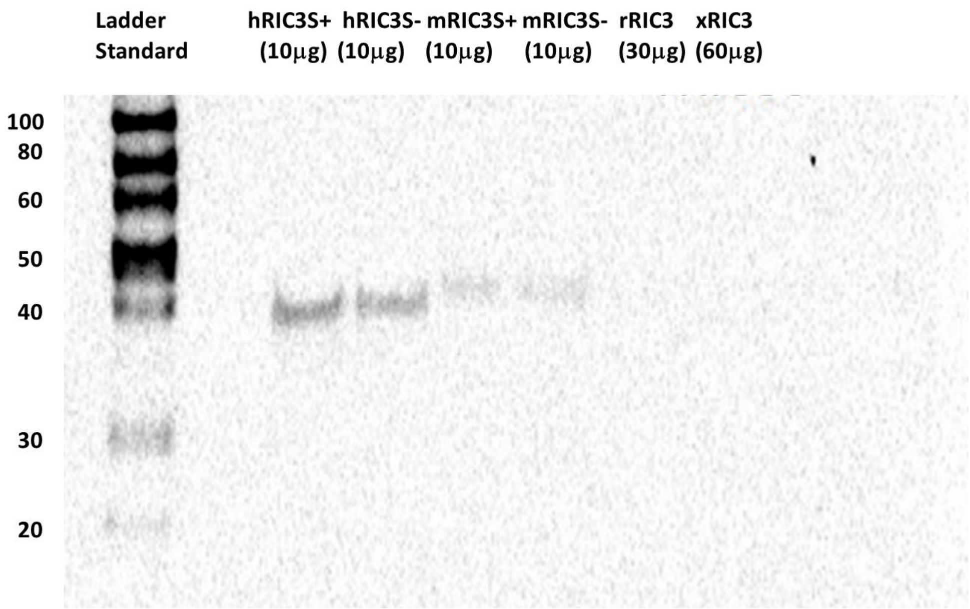

Figure S3. Novus antibody weakly stains both human and mouse RIC3 splice variants. All samples are tagged with DDK except rat RIC3. Abbreviations: hRIC3S+: Human RIC3 with the extra serine splice variant, hRIC3S-: human RIC3 lacking the serine splice variant, mRIC3S+: mouse RIC3 with the extra serine splice variant, mRIC3 S-: mouse RIC3 lacking the serine splice variant, rRIC3: rat RIC3 lacking the extra serine, xRIC3: Xenopus RIC3. All but rat RIC3 were FLAG tagged and the amount of protein loaded in each well (in parentheses) was determined to give similar RIC3 loading determined by anti-DDK antibody staining.

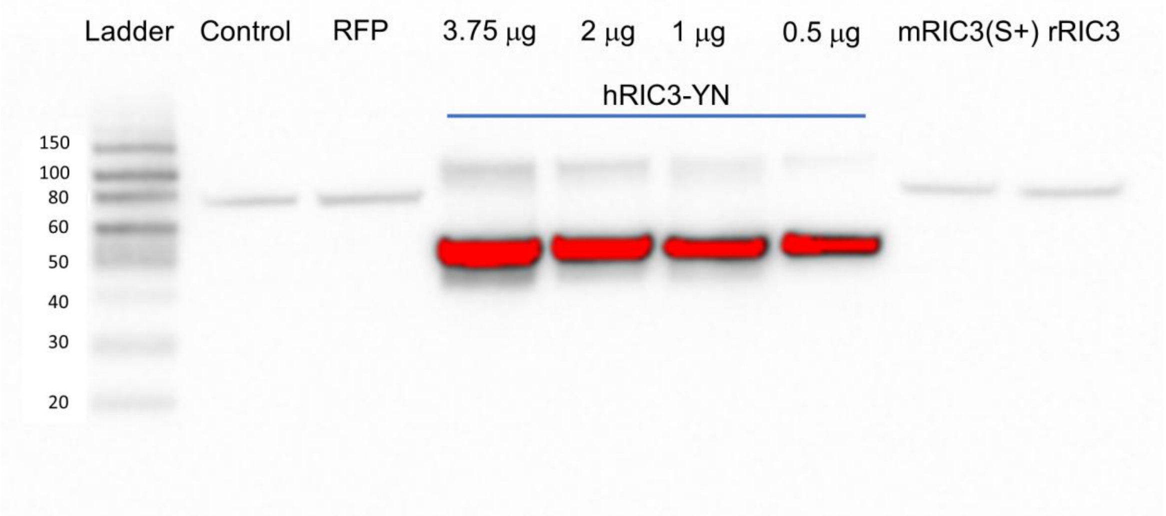

Figure S4A. In early testing, we only had human SNP variant RIC3-YN, and mouse RICS+ and rat RIC3S- splice variants. Thermo-Fisher anti-hRIC3 PA5-48432 recognizes human RIC3-YN, but not mouse or rat RIC3. It also recognizes a non-specific band in the control and other samples at around 80 kDa.

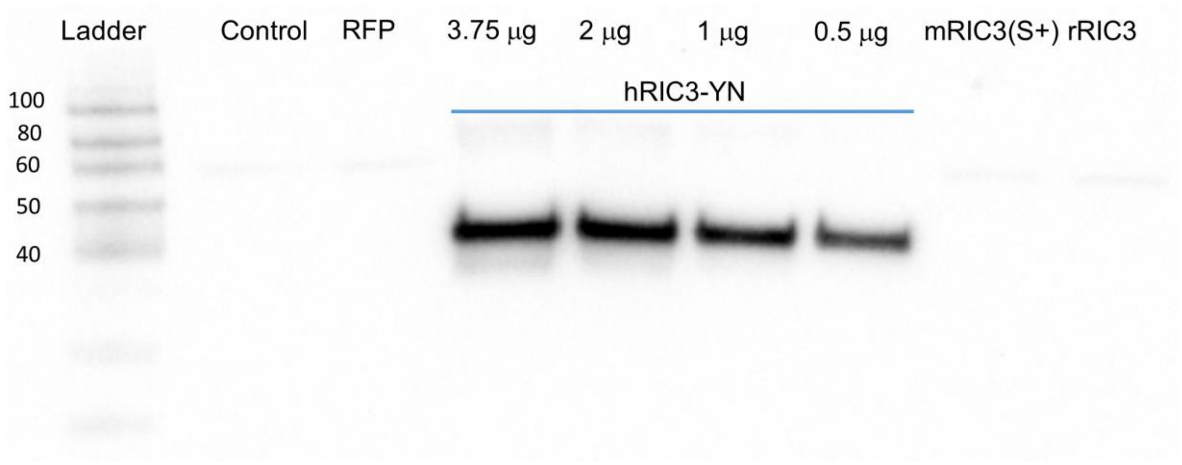

Figure S4B. Similarly, Abcam ab112911 recognizes human SNP variant RIC3-YN, but not mouse or rat RIC3

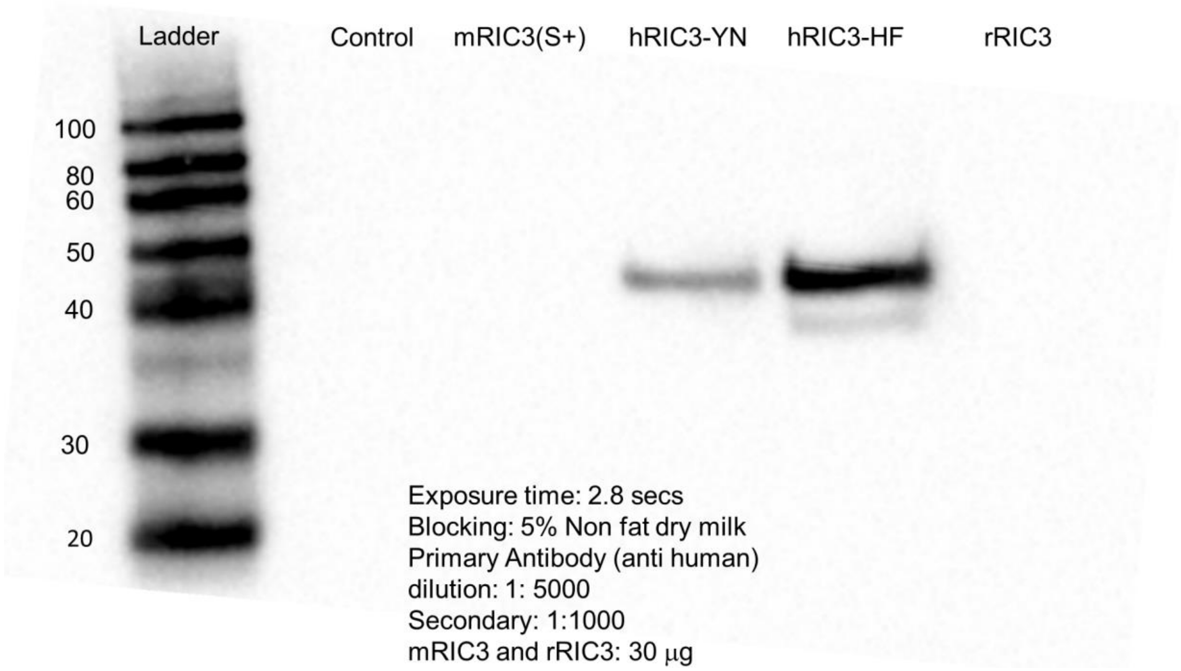

Figure S4C. Santa Cruz Biotechnology sc-377408 recognizes human RIC3-YN & human RIC3-HF SNP variants, but not mouse or rat RIC3

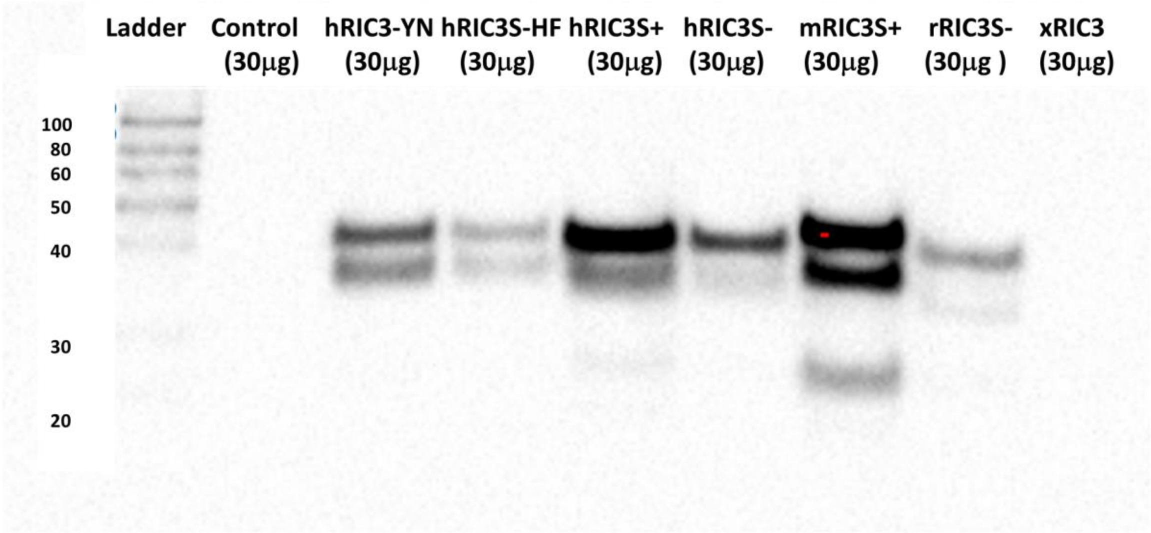

Figure S5. Thermofisher PA5-64196 (1:10,000 dilution) recognizes all human splice variants and SNPS, as well as mouse RIC3(S+) and rat(S+) RIC3, but not Xenopus. We tried high dilutions of primary antibody to see if the affinity of the antibody varied for the different constructs. There may be slightly weaker signals with SNPs and splice variants but the effect is not dramatic.

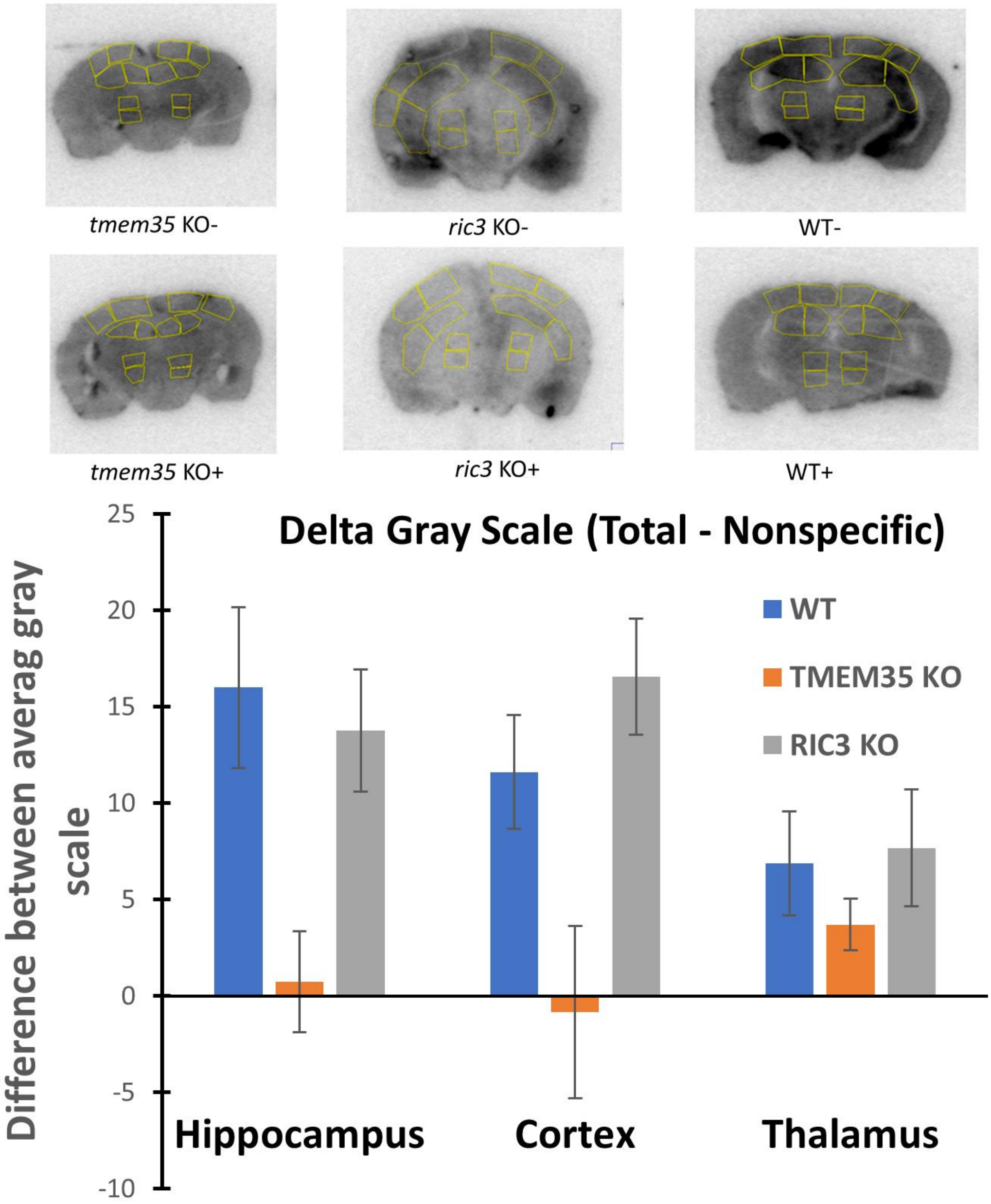

Figure S6. A second auto-radiographic experiment on sections from different animals than figure 5. Top: Representative autoradiograms of brain slices from *tmem35A* KO, *ric3* KO and Wild Type (WT) animals. The yellow lines show the different analyzed regions. A minus represents total binding (no added unlabeled toxin) while a plus indicates nonspecific binding in the presence of excess unlabeled (1 μM) toxin. Bottom: A plot of the difference between average pixel grayscales (total binding – nonspecific as determined by ImageJ) in the hippocampus, cortex and thalamus over the regions indicated above. Error bars represent the square root of the sum of the standard deviations squared for total (-) and nonspecific binding (+) for each genotype. The morphology of the sections stained with hematoxylin determined the regions chosen and were aligned with the autoradiograms using the ImageJ Align Image plugin.

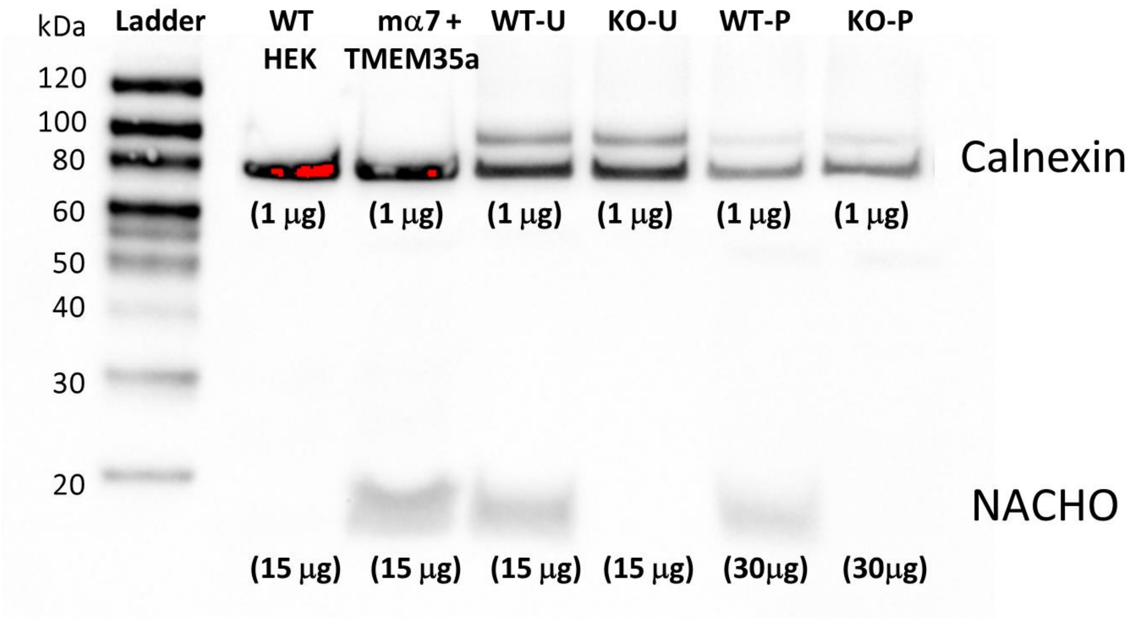

Figure S7. Immunoblots for NACHO and Calnexin from transfected HEK cells and mouse cortex. 106 HEK cells were plated in 6 well plates and were either not transfected (WT HEK) or transfected with mouse α7nAChR and mouse TMEM35a at a 1:1 ratio (m α 7 + TMEM35a). Transfections were with 250 µl of Opti-MEM® I Reduced serum-free medium, mixed with 2.5 µg DNA (1.25 µg each), 2.5 µl Plus® reagent and 10 µl of Lipofectamine LTX DNA Transfection Reagent following the manufacturer's protocol (Thermo-Fisher, Waltham MA). Four days later, the cultures were lysed with ice-cold RIPA buffer (Cell Signaling Technologies, Danvers, MA) plus protease inhibitors and then spun at 20,000 g for 10 min. Pools of 3 cortices from wild type (WT) or TMEM35a KO mice were homogenized with a "tissue tearor" (BioSpec, Bartlesville OK) in 5 ml ice-cold brain lysis buffer (0.32M sucrose, 50 mM HEPES pH7.4, 100 mM NaCl, 1 mM EDTA, 1 mM PMSF and protease inhibitor cocktail- recipe from [3]). The homogenates were centrifuged at 800 g for 10 mins and the pellets reserved for further processing. Membrane fractions in the supernatant were pelleted in an ultracentrifuge at 100,000 g for 1 hr and the pellets were solubilized in brain lysis buffer containing 1% Dodecyl-D-maltoside (U samples). The reserved pellets consisting of tissue debris from the 800 g centrifugation step were also solubilized in brain lysis buffer with 1% Dodecyl-D-maltoside (P samples). Insoluble material from each sample were removed by centrifugation at 20,000 g for 1 h. The supernatants of both cultures and brain samples were run on Western blots after determining protein concentrations.

This figure is a composite with the calnexin blot pasted over the NACHO blot using the molecular weight ladders as guides. The numbers in parentheses represent the micrograms protein applied to each well. The calnexin blot was developed with 1:3000 primary antibody (Abcam #ab22595, Cambridge MA) and 1:5000 anti-rabbit secondary (Cell Signaling Technologies #7074) for 1 sec exposure. The NACHO blot was developed with 1:1000 primary antibody (Sigma-Aldrich # HPA048583, St. Louis MO) and 1:5000 anti-rabbit (as above) with a 45 sec exposure. The nominal

molecular weight for mouse calnexin is 67 kDa, but the Abcam website notes that it runs on SDS-PAGE up to ~90 kDa due to excessive negative charges on the protein, affecting its mobility. Calnexin and NACHO are enriched in the U samples compared to the P samples since twice the protein was used to give weaker bands in the latter.

References:

38 Lukas, R.J.; Norman, S.A.; Lucero, L. Characterization of Nicotinic Acetylcholine Receptors Expressed by Cells of the SH-SY5Y Human Neuroblastoma Clonal Line. *Mol. Cell Neurosci.* **1993**, *4*, 1–12, doi:10.1006/mcne.1993.1001.

39 Peng, J.H.; Lucero, L.; Fryer, J.; Herl, J.; Leonard, S.S.; Lukas, R.J. Inducible, heterologous expression of human alpha7-nicotinic acetylcholine receptors in a native nicotinic receptor-null human clonal line. *Brain Res.* **1999**, *825*, 172–179, doi:10.1016/s0006-8993(99)01066-5.

40 Liu, C.; Shen, F.M.; Le, Y.Y.; Kong, Y.; Liu, X.; Cai, G.J.; Chen, A.F.; Su, D.F. Antishock effect of anisodamine involves a novel pathway for activating alpha7 nicotinic acetylcholine receptor. *Crit. Care Med.* **2009**, *37*, 634–641, doi:10.1097/CCM.0b013e31819598f5.

41 Williams, M.E.; Burton, B.; Urrutia, A.; Shcherbatko, A.; Chavez-Noriega, L.E.; Cohen, C.J.; Aiyar, J. Ric-3 promotes functional expression of the nicotinic acetylcholine receptor alpha7 subunit in mammalian cells. *J. Biol. Chem.* **2005**, *280*, 1257–1263, doi:10.1074/jbc.M410039200.

42 Peng, X.; Katz, M.; Gerzanich, V.; Anand, R.; Lindstrom, J. Human alpha 7 acetylcholine receptor: Cloning of the alpha 7 subunit from the SH-SY5Y cell line and determination of pharmacological properties of native receptors and functional alpha 7 homomers expressed in Xenopus oocytes. *Mol. Pharm.* **1994**, *45*, 546–554.

43 Groot Kormelink, P.J.; Luyten, W.H. Cloning and sequence of full-length cDNAs encoding the human neuronal nicotinic acetylcholine receptor (nAChR) subunits beta3 and beta4 and expression of seven nAChR subunits in the human neuroblastoma cell line SH-SY5Y and/or IMR-32. *FEBS Lett.* **1997**, *400*, 309–314, doi:10.1016/s0014-5793(96)01383-x.

44 Charpantier, E.; Wiesner, A.; Huh, K.H.; Ogier, R.; Hoda, J.C.; Allaman, G.; Raggenbass, M.; Feuerbach, D.; Bertrand, D.; Fuhrer, C. Alpha7 neuronal nicotinic acetylcholine receptors are negatively regulated by tyrosine phosphorylation and Src-family kinases. *J. Neurosci.* **2005**, *25*, 9836–9849, doi:10.1523/JNEUROSCI.3497-05.2005.

45 Kuryatov, A.; Mukherjee, J.; Lindstrom, J. Chemical chaperones exceed the chaperone effects of RIC-3 in promoting assembly of functional alpha7 AChRs. *PLoS ONE* **2013**, *8*, e62246, doi:10.1371/journal.pone.0062246.

46 Sweileh, W.; Wenberg, K.; Xu, J.; Forsayeth, J.; Hardy, S.; Loring, R.H. Multistep expression and assembly of neuronal nicotinic receptors is both host-cell- and receptor-subtype-dependent. *Brain Res. Mol. Brain Res.* **2000**, *75*, 293–302, doi:10.1016/s0169-328x(99)00302-2.

47 Yue, Y.; Liu, R.; Cheng, W.; Hu, Y.; Li, J.; Pan, X.; Peng, J.; Zhang, P. GTS-21 attenuates lipopolysaccharide-induced inflammatory cytokine production in vitro by modulating the Akt and NF-kappaB signaling pathway through the alpha7 nicotinic acetylcholine receptor. *Int. Immunopharmacol.* **2015**, *29*, 504–512, doi:10.1016/j.intimp.2015.10.005.
